# Efficacy of Intravenous Tranexamic Acid in Reducing Perioperative Blood Loss and Blood Product Transfusion Requirements in Patients Undergoing Multilevel Thoracic and Lumbar Spinal Surgeries: A Retrospective Study

**DOI:** 10.3389/fphar.2020.566956

**Published:** 2020-11-30

**Authors:** Alexandre B. Todeschini, Alberto A. Uribe, Marco Echeverria-Villalobos, Juan Fiorda-Diaz, Mahmoud Abdel-Rasoul, Benjamin G. McGahan, Andrew J. Grossbach, Stephanus Viljoen, Sergio D. Bergese

**Affiliations:** ^1^ Department of Anesthesiology, Wexner Medical Center, The Ohio State University College of Medicine, Columbus, OH, United States; ^2^ Center for Biostatistics, Wexner Medical Center, The Ohio State University College of Medicine, Columbus, OH, United States; ^3^ Department of Neurological Surgery, Wexner Medical Center, The Ohio State University College of Medicine, Columbus, OH, United States; ^4^ Department of Anesthesiology, Stony Brook University, Stony Brook, NY, United States

**Keywords:** neurosurgery, spinal surgery, blood loss, tranexamic acid, spinal fusion, blood transfusion

## Abstract

**Introduction:** Acute perioperative blood loss is a common and potentially major complication of multilevel spinal surgery, usually worsened by the number of levels fused and of osteotomies performed. Pharmacological approaches to blood conservation during spinal surgery include the use of intravenous tranexamic acid (TXA), an anti-fibrinolytic that has been widely used to reduce blood loss in cardiac and orthopedic surgery. The primary objective of this study was to assess the efficacy of intraoperative TXA in reducing estimated blood loss (EBL) and red blood cell (RBC) transfusion requirements in patients undergoing multilevel spinal fusion.

**Materials and Methods:** This a single-center, retrospective study of subjects who underwent multilevel (≥7) spinal fusion surgery who received (TXA group) or did not receive (control group) IV TXA at The Ohio State University Wexner Medical Center between January 1st, 2016 and November 30th, 2018. Patient demographics, EBL, TXA doses, blood product requirements and postoperative complications were recorded.

**Results:** A total of 76 adult subjects were included, of whom 34 received TXA during surgery (TXA group). The mean fusion length was 12 levels. The mean total loading, maintenance surgery and total dose of IV TXA was 1.5, 2.1 mg per kilo (mg/kg) per hour and 33.8 mg/kg, respectively. The mean EBL in the control was higher than the TXA group, 3,594.1 [2,689.7, 4,298.5] vs. 2,184.2 [1,290.2, 3,078.3] ml. Among all subjects, the mean number of intraoperative RBC and FFP units transfused was significantly higher in the control than in the TXA group. The total mean number of RBC and FFP units transfused in the control group was 8.1 [6.6, 9.7] and 7.7 [6.1, 9.4] compared with 5.1 [3.4, 6.8] and 4.6 [2.8, 6.4], respectively. There were no statistically significant differences in *postoperative* blood product transfusion rates between both groups. Additionally, there were no significant differences in the incidence of 30-days postoperative complications between both groups.

**Conclusion:** Our results suggest that the prophylactic use of TXA may reduce intraoperative EBL and RBC unit transfusion requirements in patients undergoing multilevel spinal fusion procedures ≥7 levels.

## Introduction

Multilevel spinal fusion surgery is among the most common procedures (ranked fifth in 2017) performed on inpatients in the United States (Healthcare Cost and Utilization Project (HCUP), 2017; Goobie et al., 2018; Verma et al., 2010). Acute perioperative blood loss is a common and potentially major complication of multilevel spinal surgery, usually worsened by the number of levels fused and of osteotomies performed (Lin et al., 2018; Lu et al., 2018; Shakeri et al., 2018). For cases involving more levels, the larger operative wound and area of bleeding surfaces exposed have been associated with increased blood loss (Nagabhushan et al., 2018).

There are several patient factors that contribute to the significant intraoperative blood loss in spinal fusion surgery including, but not limited to: height of the patient, length of surgical field exposure and severity or type of spine deformity (Verma et al., 2010). Additionally, there are surgical factors that include: length of surgery, type of procedure, combined approaches (anterior and posterior), number of levels fused, number of anchors placed, intraoperative maintenance of the mean arterial pressure, utilization of blood salvage techniques, history of coagulopathy and the use of anti-fibrinolytic drugs (Verma et al., 2010). Also, the prone position might lead to hemodynamic challenges during surgery by restricting the blood flow of the inferior vena cava and consequently distending the paravertebral and epidural veins, which contributes to increased bleeding (DePasse et al., 2015). In order to minimize this, the use of the Jackson table has been recommended due to its capacity to reduce intra-abdominal pressure (Jackson, 1992; Mathai et al., 2012).

The prevention of blood loss is crucial to spinal surgery since massive blood loss can contribute to coagulopathy and disseminated intravascular coagulation, increasing the risk of postoperative hematoma and neurological deficits (Hu, 2004; Elgafy et al., 2010).

Traumatic hemorrhage, as well as profuse intraoperative bleeding, promotes massive release of tissue plasminogen activator (tPA) inducing hyperfibrinolysis (Kashuk et al., 2010; Chapman et al., 2016). Plasminogen, produced by the liver, and tPA bind to C-terminal lysine residues on fibrin leading to localized plasmin formation and fibrin cleavage (Chapman et al., 2016). Tranexamic Acid (TXA) is an anti-fibrinolytic that has been widely used to prevent massive blood loss in cardiac and orthopedic surgery, with an increased use in spinal surgeries. (Verma et al., 2010; Hunt, 2015; Yoo et al., 2019). TXA inhibits the capacity of plasminogen and plasmin to bind to fibrin, hence preserving blood clots from plasmin-mediated lysis (Verma et al., 2010; Hunt, 2015). TXA, a synthetic derivative of lysine, exerts its antifibrinolytic activity by reversibly and competitively binding to lysine-binding sites on the structural proteins of plasmin, plasminogen, and as well as tPA, this consequently inhibits the degradation of fibrin molecules (McCormack, 2012). Each plasminogen molecule has up to five binding sites for TXA, with one site of high affinity (McCormack, 2012). Data from animal models of traumatic injuries also suggests a delayed release of urokinase plasminogen activator (uPA) (Hijazi et al., 2015). Physiologically, TXA catalyzes the conversion of plasminogen to active plasmin, which induces controlled anticoagulation and clot breakdown (Hijazi et al., 2015). There is evidence showing that TXA has pharmacological actions other than inhibition of fibrinolysis (Couturier and Grassin-Delyle, 2014), such as clot stabilization (Dai et al., 2011), improvement of platelet function (Blake et al., 2006), inhibition of apoptosis (Hsia et al., 2010), and prevention of pro-inflammatory cytokine production (Jimenez et al., 2007). However, its safety and efficacy profiles are not well known in spinal surgery (Lin et al., 2018; Lu et al., 2018; Luo et al., 2018; Nagabhushan et al., 2018; Shakeri et al., 2018; Xue et al., 2018; Karimi et al., 2019).

The literature describes different volume, timing and route of TXA administration for spinal procedures. Intravenous administration is the most Common, ranging from 10–20 mg per kilogram (mg/kg) as a loading dose and 1–10 mg per kilogram per hour (mg/kg/h) as a maintenance dose (Walterscheid et al., 2017). Similar to its use in other surgical procedures, the most common side effects related to TXA are seizures, acute kidney injury, liver injury, and mainly thromboembolic events (myocardial infarction, stroke, deep vein thrombosis and pulmonary embolism), particularly in elderly patients (Neilipovitz et al., 2001; Verma et al., 2010; Colomina et al., 2017; Grassin-Delyle et al., 2018; Nagabhushan et al., 2018). The efficacy of its use has been assessed in spinal surgery and the results are inconclusive due to the complexity and diversity of the dosing regimen (Neilipovitz et al., 2001; Wong et al., 2008; Verma et al., 2010; Farrokhi et al., 2011; Colomina et al., 2017; Johnson et al., 2017; Carabini et al., 2018; Grassin-Delyle et al., 2018; Lin et al., 2018; Lu et al., 2018; Nagabhushan et al., 2018; Karimi et al., 2019). After the consideration of intraoperative blood loss and the incidence of autologous transfusion, the benefit of using TXA might outweigh the undesirable side effects. However, although it is widely used, it is not currently considered part of standard of care in spinal surgeries (Verma et al., 2010; Walterscheid et al., 2017).

The efficacy profile of TXA in spinal procedures remains unclear and should be investigated. Therefore, we designed a retrospective study with the hypothesis that patients undergoing primary or revision spinal fusion surgery (≥7 levels) who received intravenous (IV) TXA would have a significant reduction in intraoperative estimated blood loss (EBL) and red blood cell (RBC) transfusions compared with patients who did not receive IV TXA. The primary objective of the study was to assess the efficacy of intraoperative TXA in reducing EBL and RBC transfusions in patients undergoing multilevel spinal fusion.

## Materials and Methods

### Research Design

We conducted a single-center, retrospective study that reviewed electronic medical records from subjects who underwent multilevel (≥7) spinal fusion surgery and received (TXA group) or did not receive (control group) IV TXA at The Ohio State University Wexner Medical Center between January 1st, 2016 and November 30th, 2018. The decision of dosing TXA during surgery was at the surgeon’s discretion. After obtaining the approval of our Institutional Review Board (Office of Responsible Research Practices, The Ohio State University), we accessed electronic medical records in order to assess eligibility and collect perioperative information from 404 subjects.

### Participants

Subjects 18 years and older that underwent ≥7 levels spinal fusion surgery (primary or revision) using a posterior midline approach and received loading and maintenance dose TXA between January 1st, 2016 and November 30th, 2018 at The Ohio State University Wexner Medical Center, Department of Neurosurgery, were included in the study. Prisoners, pregnant women, history of congenital coagulation disorders, chronic kidney disease (baseline serum creatinine >1.36 mg per deciliter [mg/dl]), chronic liver disease, concomitant use of coagulation-altering medications, or undergoing active treatment for malignancies were excluded from the study.

### Study Procedures

The following information was collected for analysis purposes: demographics (age, gender, race, height, weight, BMI), the American Society of Anesthesiology (ASA) physical status, perioperative coagulation and hematologic parameters, surgical variables (number of levels fused, length of surgery and of anesthesia, previous spinal surgery, spinal surgeon, type of spinal pathology, type of surgical technique, EBL, units, volume and type of blood transfusions administered (considering that the threshold for blood products transfusion was hemoglobin ≤7 mg/dl), total dose of IV TXA administration, length of post-anesthesia care unit (PACU) and hospital stay. In addition, type and units of blood product transfusions administered during the first 24 h after the end of surgery and any complications diagnosed in a 30-days period after surgery were also collected. Intraoperative transfusions were performed in accordance with our institutional transfusion protocol (“Transfusion Therapy: Indications for Ordering”). If the patient was hemodynamically stable, intraoperative blood transfusion was considered at a hemoglobin threshold of <8 gm/dl, if intraoperative bleeding is not expected to be life-threaten. In addition, the decision to transfuse blood products during surgery was made by the anesthesiology care provider following our institutional transfusion protocol. Consequently, as part of our institutional transfusion protocol, platelets can be ordered for pooled (4 or 6 units) prior to surgery and if more units are required, single units are dispensed and all blood products used at our institution are leukoreduced. In addition, the intraoperative estimation of EBL was made according to the standard procedure as the difference between the total surgical suctioned volume and the total amount of irrigation used during the case. The hemoglobin concentration was measured in all patients preoperatively. All gauzes and lap sponges were wringed into the field, circulated through the suction device, and accounted for by EBL. Postoperative EBL was estimated by postoperative drain output.

### Outcomes

The primary outcomes were incidence and quantity of intraoperative EBL and RBC transfusions. Our secondary outcomes were the incidence and quantity of other perioperative blood product transfusion requirements, surgical variables (length of surgery, anesthesia, PACU, intensive care unit (ICU) and hospitalization stay) the incidence of 30-days postoperative complications in both groups.

### Statistical Methods

Continuous patient demographic and clinical/surgical variables were summarized as means (±standard deviations) and categorical variables were summarized as frequencies (percentages). Student’s t-tests or Mann-Whitney U tests were used to test demographic differences between study groups for continuous variables where appropriate. Chi-square or Fisher’s exact tests were used to test for differences in categorical variables between study groups where appropriate. Linear regression models were fit for each of the outcomes in the primary aim adjusting for potential confounders and effect modifiers, including number of spinal levels, cell saver use and number of surgeons variable. The final models for RBC and FFP included main effects for study group and number of spine levels. The final model for EBL also included an interaction term between study group and number of spine levels. All models were checked to verify that statistical assumptions of normality of residuals and equal variance were not violated. Hypothesis testing comparing outcomes between study groups was administered based on the final model estimates for each outcome and conducted at a two-sided alpha of 0.05. All statistical analyses were performed using SAS/STAT software version 9.4 (SAS Institute Inc., Cary, NC).

## Results

**FIGURE 1:**
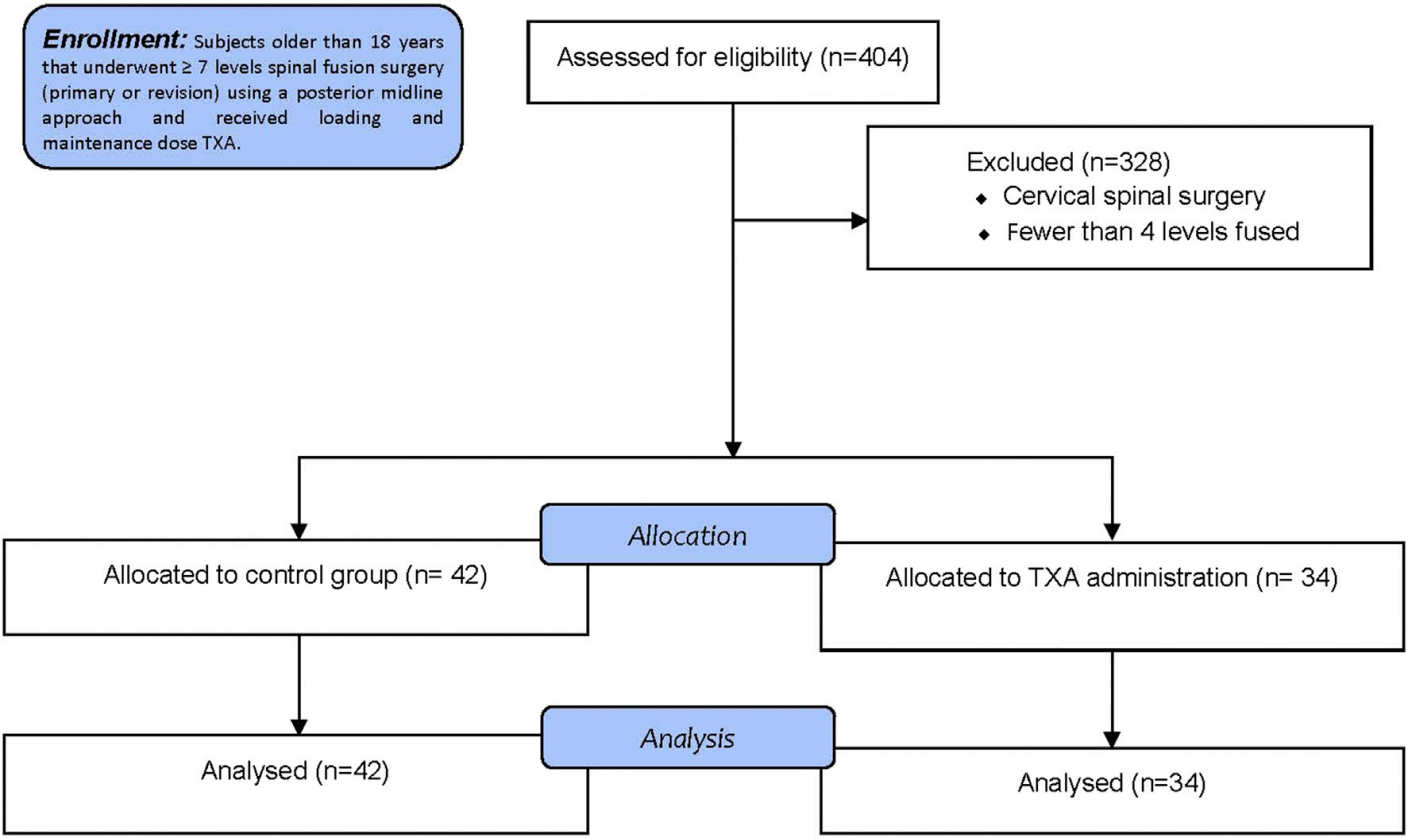
Retrospective analysis consort flow diagram. N, number; TXA, tranexamic acid.

A total of 404 charts were initially reviewed. Data from 328 subjects were not eligible based on inclusion and exclusion criteria; the main reasons for exclusion were cervical spinal surgeries, fewer than seven levels fused and minimally invasive procedures. Therefore, this study included 76 subjects for data analysis, of whom 42 (55.3%) did not receive IV TXA (control group) and 34 (44.7%) received IV TXA (TXA group) during surgery. The flow-diagram of this retrospective observational trial according to the Consolidated Standards of Reporting Trials (CONSORT) is shown in Figure 1 (Begg et al., 1996).

A summary of demographic and perioperative variables is listed in Table 1. The mean age was 59.3 (±12.0) years in the control group and 58 (±12.0) years in the TXA group. The mean total loading dose, dose rate for maintenance and total dose administered during maintenance of IV TXA administration was 1.5 (±0.9) mg, 2.1 (±1.3) mg/kg/h and 33.8 (±20.3) mg/kg, respectively. There was not a statistically significant difference in demographic and clinical characteristics except for length of anesthesia and surgery. Subjects in the TXA group had a significantly shorter mean length of anesthesia (9.8 ± 1.8 vs. 10.9 ± 2.3 h; *p*-value = 0.0296) and surgery (7.2 ± 1.6 vs. 8.3 ± 2.3 h; *p*-value = 0.0227).

**TABLE 1:** Demographic and perioperative variables.

Demographic and perioperative variables	Overall (n = 76)	Control (n = 42)	TXA (n = 34)	*p*-value
Age, years, mean (SD)	58.8 (11.9)	59.3 (12.0)	58 (12.0)	0.6383
Sex, male, n (%)	37 (48.7)	17 (40.5)	20 (58.8)	0.1116
Sex, female, n (%)	39 (51.3)	25 (59.5)	14 (41.2)	
Height, cm, mean (SD)	168.9 (11.0)	168.7 (11.4)	169.1 (10.6)	0.8695
Weight, kg, mean (SD)	85.4 (23.8)	85 (25.5)	85.8 (21.7)	0.8713
BMI, kg/m^2^, mean (SD)	29.7 (7.0)	29.6 (7.7)	29.8 (6.2)	0.9074
ASA classification, I/II/III/IV, n	0/19/55/2	0/8/33/1	0/11/22/1	0.4233
Number of fusion levels, mean (SD)	12.3 (3.4)	12.5 (3.5)	12.0 (10.8)	0.5289
Length of anesthesia, hours, mean (SD)	10.4 (2.1)	10.9 (2.3)	9.8 (1.8)	**0.0296**
Length of surgery, hours, mean (SD)	7.8 (2.1)	8.3 (2.3)	7.2 (1.6)	**0.0227**
ICU admission, n (%)	71 (93.4)	38 (90.5)	33 (97.1)	0.3725
Cell saver used, n, (%)	8 (10.5)	6 (14.3)	2 (5.9)	0.2853
ICU length of stay, days, mean (SD)	3.1 (2.9)	3.1 (3.2)	3.1 (2.5)	0.9713
Length of hospital stay, days, mean (SD)	8.8 (3.6)	8.8 (3.1)	8.7 (4.2)	0.8478
Surgeon A, n (%)	60 (78.9%)	31 (73.8)	29 (85.3)	0.22
Previous spine surgery	35 (46.1)	21 (50)	14 (41.18)	0.4429
Pathology type				
Fracture	6 (7.9)	4 (9.52)	2 (5.88)	0.6858
Tumor	0	0	0	NA
Degenerative	26 (34.2)	12 (28.6)	14 (41.2)	0.2494
Deformity	52 (68.4)	29 (69.1)	23 (67.7)	0.8961
Spinal stenosis	33 (43.4)	21 (50)	12 (35.3)	0.1984
Foraminal stenosis	15 (19.7)	6 (14.29)	9 (26.47)	0.1845
Type of surgical technique				
Complex fusion	76 (100)	42 (55.0)	34 (45.0)	NA
Decompression	50 (65,8)	28 (66.67)	22 (64.71)	0.8578
Osteotomy	76 (100)	42 (55.0)	34 (45.0)	NA
Baseline hematologic and coagulation tests				
Hb, g/dL, mean (SD)	13.2 (1.9)	13.0 (1.7)	13.5 (2.0)	0.314
Ht, %, mean (SD)	40.2 (5.2)	39.6 (5.0)	40.8 (5.4)	0.3173
Platelets, 10^3^/L, mean (SD)	247.7 (73.7)	244.3 (68.8)	251.8 (80.1)	0.6638
PT, s, mean (SD)	13.5 (1.8)	13.3 (0.9)	13.8 (2.5)	0.2864
PTT, s, mean (SD)	29.9 (4.2)	29.7 (3.9)	30.2 (4.6)	0.5477
INR, mean (SD)	1.1 (0.2)	1.0 (0.1)	1.1 (0.3)	0.2593
TXA total loading dose, g, overall, mean (SD)			1.5 (0.9)	
TXA dose rate, mg/kg/h, overall, mean (SD)			2.1 (1.3)	
TXA total dose, mg/kg, overall, mean (SD)			33.8 (20.3)	

N, number; %, percentage; cm, centimeter; SD, standard deviation; kg, kilogram; BMI, body mass index; m2, meter square; ICU, intensive care unit; ASA, American Society of Anesthesiology physical status classification; Hb, hemoglobin; g/dL, Gram per deciliter; L, liter; ht, hematocrit; PT, prothrombin time; s, seconds; PTT, Partial Thromboplastin Time; INR, international Normalized Ratio; TXA, tranexamic acid; g, Gram; mg/kg/hr, milligram per kilogram per hour; mg/Kg, milligram per kilogram.

The mean EBL in the control was higher than the TXA group, 3,494.1 [2,689.7, 4,298.5] vs. 2,184.2 [1,290.2, 3,078.3] ml (95% confidence interval [CI]; *p*-value = 0.0332) (Table 2). Among all subjects, the mean number of intraoperative RBC and FFP units transfused was significantly higher (95% CI; *p*-value < 0.05) in the control group than in the TXA group. The total mean number of RBC and FFP units given to subjects in the control group was 8.1 [6.6, 9.7] and 7.7 [6.1, 9.4] compared with 5.1 [3.4, 6.8] and 4.6 [2.8, 6.4], respectively (Table 2).

**TABLE 2:** Estimated blood loss and amount of intraoperative blood transfusion from univariate models.

Blood Products and EBL	Control (n = 42)	TXA (n = 34)	*p*-value
EBL, ml, overall, mean (95% CI)	3,494.07 (2,689.68, 4,298.46)	2,184.24 (1,290.21, 3,078.26)	**0.0332**
Blood product transfusions			
RBC, units, overall, mean (95% CI)	8.12 (6.57, 9.67)	5.09 (3.37, 6.81)	0.0110
FFP, units, overall, mean (95% CI)	7.71 (6.08, 9.35)	4.59 (2.77, 6.40)	0.0128

EBL, estimated blood loss; n, number; TXA, tranexamic acid; %, percentage; CI, confidence interval; RBC, red blood cells; FFP, fresh frozen plasma.

**FIGURE 2:**
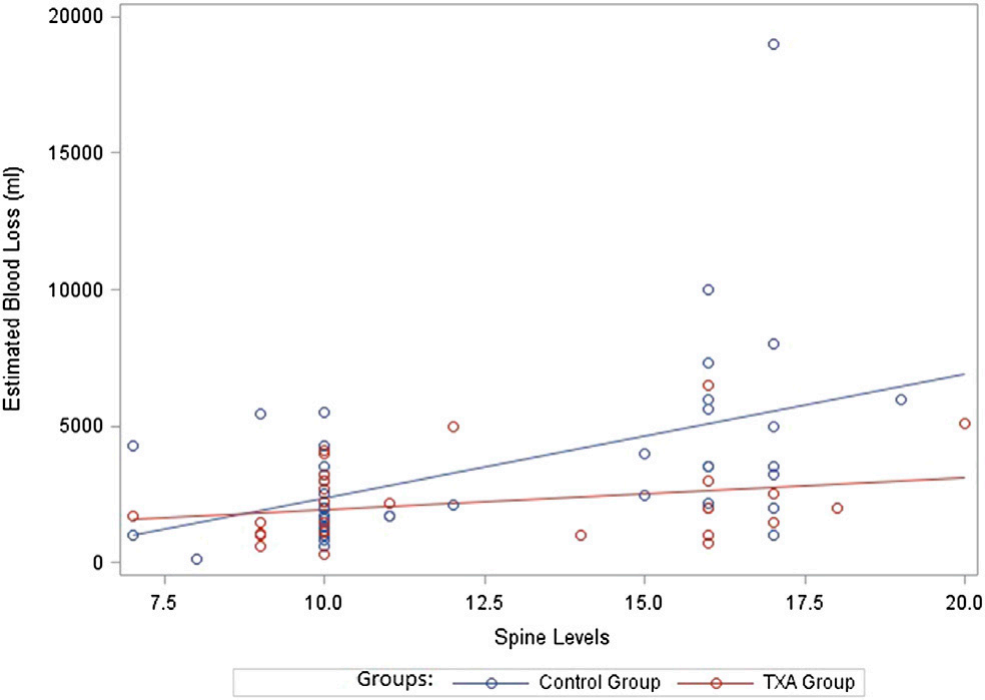
Estimated blood loss. ml, milliliters; TXA, tranexamic acid.

**FIGURE 3:**
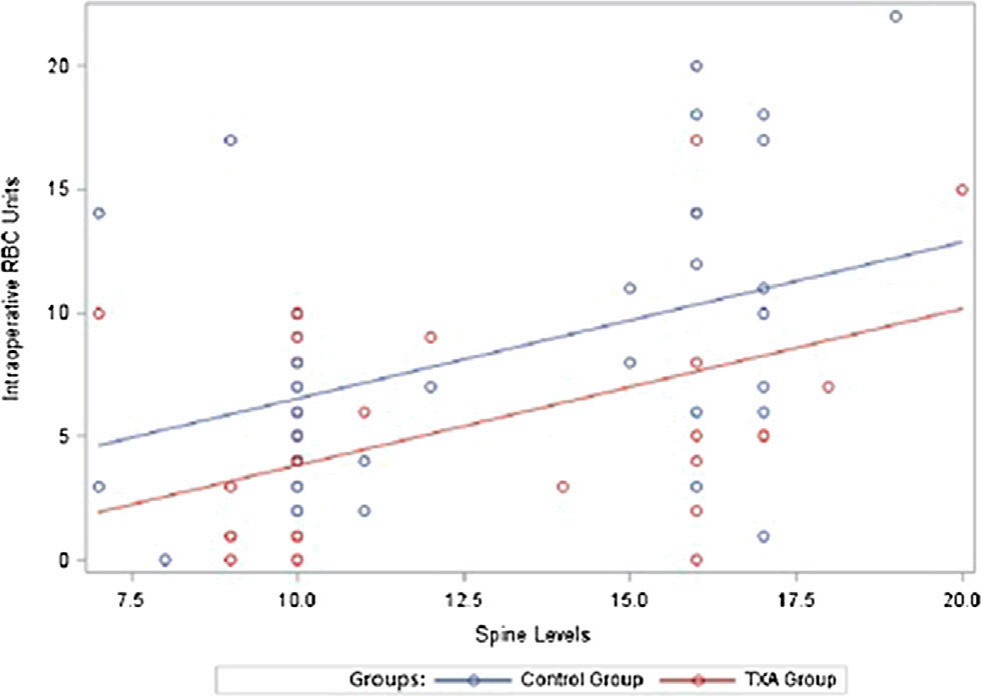
Intraoperative transfused red blood cell units. RBC, red blood cells; TXA, tranexamic acid.

**FIGURE 4:**
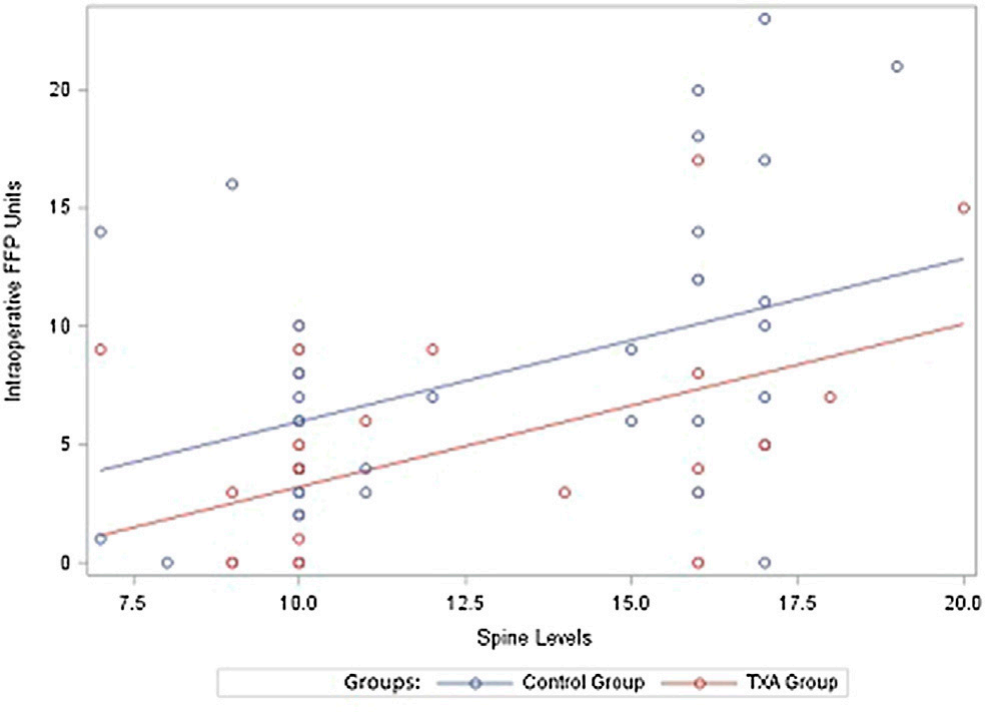
Intraoperative transfused frozen fresh plasma units. FFP, fresh frozen plasma; TXA, tranexamic acid.

A multivariate model predicting EBL was fit including main effects for study group, number of spine levels and the interaction between the two variables. EBL increased at a greater rate for the control group compared to the TXA group as number of spine levels increased (interaction term *p*-value = 0.0386) (Table 3; Figure 2). Additionally, a multivariate model predicting RBC transfusion was fit including main effects for study group and number of spine levels. RBC transfusion was significantly higher on average for the control group compared to the TXA group (*p*-value = 0.0123). There was also a significant increase in blood loss as number of spine levels increased (*p*-value = 0.0001). An interaction term between study group and the number of spine levels was not included in the final multivariable RBC model as it was not statistically significant (*p*-value = 0.1708) (Table 3; Figure 3). Ultimately, a multivariate model predicting FFP transfusion was fit including main effects for study group and number of spine levels. FFP was significantly higher for the control group compared to the TXA group (*p*-value = 0.0143) after adjusting for number of spine levels. An interaction term between study group and number of spine levels was not included in the final multivariable FFP administration model as it was not statistically significant (*p*-value = 0.1697) (Table 3; Figure 4).

**TABLE 3:** Estimated blood loss and amount of intraoperative blood transfusion from multivariate models.

Blood Products and EBL	Control (n = 42)	TXA (n = 34)	*p*-value	Interaction with no. of spine levels
EBL,^a^ mL, overall, mean (95% CI)	3,391.64 (2,666.94, 4,116.35)	2,217.01 (1,410.50, 3,023.54)	**0.0341**	**0.0386**
Blood product transfusions				
RBC,^b^ units, overall, mean (95% CI)	7.98 (6.57, 9.38)	5.26 (3.70, 6.83)	0.0123	
FFP,^b^ units, overall, mean (95% CI)	7.56 (6.09, 9.04)	4.78 (3.14, 6.42)	0.0143	

EBL, estimated blood loss; n, number; TXA, tranexamic acid; %, percentage; CI, confidence interval; RBC, red blood cells; FFP, fresh frozen plasma.

a Adjusted of no. of spine levels and interaction of spine levels and study group.

b Adjusted for no. of spine levels.

Subsequently, there was no statistical difference in *postoperative* blood product transfusion rates between both groups. As a final point, there was no significant difference in the incidence of 30-days postoperative complications between both groups (Table 4).

**TABLE 4:** Postoperative complications.

Post-operative complication (at 30 days)	Overall (N = 76)	Control (N = 42)	TXA (N = 34)	*p*-value
Incidence of complications, n, (%)	35 (46.05)	19 (45.24)	16 (47.06)	0.8742
Complications list, n, (%)				
Reoperations	5 (6.58)	3 (7.14)	2 (5.88)	>0.9999
Readmissions	11 (14.47)	4 (9.52)	7 (20.59)	0.1728
Wound infection	1 (1.32)	1 (2.38)	0 (0.00)	>0.9999
Pulmonary embolism	2 (2.63)	0 (0.00)	2 (5.88)	0.1968
Deep vein thrombosis	3 (3.95)	2 (4.76)	1 (2.94)	>0.9999
Seizures	1 (1.32)	1 (2)	0 (0.00)	>0.9999
Myocardial infarction	0 (0.00)	0 (0.00)	0 (0.00)	—
Stroke	0 (0.00)	0 (0.00)	0 (0.00)	—
Acute kidney injury	3 (3.95)	2 (4.76)	1 (2.94)	>0.9999
Hepatic dysfunction	0 (0.00)	0 (0.00)	0 (0.00)	—
Others	25 (32.89)	12 (28.57)	13 (38.24)	0.4634
Death	0 (0.00)	0 (0.00)	0 (0.00)	>0.9999

N, number; TXA, tranexamic acid; %, percentage.

## Discussion

In the present retrospective study, 76 subjects underwent multilevel (≥7 levels) spinal fusion and 34 of them received intraoperative loading and maintenance doses of TXA. Both groups were similar in the demographics and surgical procedure. The primary objective of the study was to assess the efficacy of intraoperative TXA in reducing EBL and RBC transfusions in patients undergoing multilevel (≥7 levels) spinal fusion. The results have shown significant difference in both groups; EBL during surgery and the number of intraoperative RBC transfused was reduced by 37% with the use of IV TXA. There was also a significant difference in the number of intraoperative RBC and FFP units transfused with the need of intraoperative transfusion being reduced by 37% and 40% in the TXA group compared to the control group (*p*-value = 0.0123 and 0.0143). A mean IV TXA loading and maintenance dose of 1.5 g and 2.1 mg/kg/h was well tolerated by subjects in the TXA group and there were no significant differences in the postoperative complications associated with its use. The results from this study will serve to direct the design and power analysis of a prospective double-blind randomized clinical trial assessing efficacy of using TXA administration in patients undergoing multilevel spinal fusion.

Complex multilevel spinal decompression, fusion and osteotomies have been associated with profuse perioperative blood loss and greater use of blood transfusions with subsequent increased risk of coagulation impairment, blood-borne infectious disease transmission, postoperative hematoma formation, shock and pulmonary edema (Johnson et al., 1989; Mirza et al., 2008; Naik et al., 2017). Studies describing the impact of perioperative TXA administration on blood loss and blood transfusion may vary among institutions. Choi et al. reviewed 132 consecutive patients (adults and children) undergoing multilevel (≥5 levels) posterior spinal instrumented fusion (Choi et al., 2017). TXA was administered in 67% of the patients (n = 89). For adult patients, an IV TXA loading dose of 10 mg/kg was used, followed by a continuous maintenance infusion dose of 1 mg/kg/h (Choi et al., 2017). In pediatrics (<18 years old), IV TXA 5 mg/kg/h. was used for maintenance following a 50 mg/kg loading dose (Choi et al., 2017). A significant reduction in EBL (841 vs. 1,396 ml; *p* = 0.002), intra- and postoperative transfusion volume (544 vs. 812 ml; *p* = 0.012 and 193 vs. 359 ml; *p* = 0.034, respectively) was reported in patients who received TXA when compared to patients in which TXA was not used (Choi et al., 2017). A multiple regression analysis demonstrated an association between TXA administration and decreased surgical bleeding (Choi et al., 2017).

Loading TXA doses are highly variable among published randomized clinical trials (RCTs) conducted in patients undergoing spinal surgeries, whereas infusion or maintenance doses seem to be more consistent. Moreover, reported intra- and postoperative outcomes may also vary. Shakeri et al. studied the efficacy of a single TXA dose of 15 mg/kg compared to placebo in 50 patients undergoing laminectomy (≥2 levels) with postero-lateral fusion. A significant reduction in mean total amount of bleeding (632.2 vs. 1,037 ml; *p* = 0.0001), RBC transfusion required (440 vs. 1,320 ml; *p* = 0.0001) and length of hospital stay (2.28 vs. 3.36 days, *p* = 0.001) was reported in patients who received TXA (Shakeri et al., 2018).

In a multicenter study, Colomina et al. randomized 95 patients undergoing posterior spinal surgery to receive either IV TXA (10 mg/kg infusion in 20 min before surgical incision followed by a 2 mg/kg/h until closure) or matched saline solution (Colomina et al., 2017). Patients undergoing thoracic or lumbar spinal fusions (>3 levels) were included in this study. Posterior and posterolateral techniques, spinal osteotomies, posterior lumbar interbody fusion (PLIF), transforaminal lumbar interbody fusion (TLIF) and instrumented procedures of at least four vertebrae (i.e. wires, pedicle screws) were some of the types of surgeries included (Colomina et al., 2017). The mean intraoperative and total blood loss was significantly reduced in patients receiving TXA when compared to placebo (*p* = 0.01) (Colomina et al., 2017). However, total number of RBC transfused was similar between groups (0.85 vs. 1.42 respectively, *p* = 0.06) (Colomina et al., 2017). Conversely, Carabini et al. reported a significant reduction in RBC transfused in patients receiving TXA (loading dose 10 mg/kg followed by 1 mg/kg/h maintenance infusion) for complex spinal fusions when compared to matched placebo (1,140 ml vs. 1,460 respectively, *p* = 0.034). Moreover, TXA use was also associated with significant decreased cell saver transfusion (256 vs. 490 ml respectively, *p* = 0.042) (Carabini et al., 2018).

A meta-analysis of 11 RCTs published in 2015 by Cheriyan et al. reported variability on TXA doses from 1 to 15 mg/kg among 644 patients undergoing spinal surgeries (Cheriyan et al., 2015). A significant reduction in intra- and postoperative blood loss was associated with TXA administration (Cheriyan et al., 2015). Moreover, total perioperative blood loss was significantly lowered in patients receiving TXA when compared to placebo (*p*-value <0.0001) with no increase in the incidence of thromboembolic events among trials (Cheriyan et al., 2015).

Another systematic review and meta-analysis recently published in 2019 by Zhang et al. assessed the influence of TXA on blood loss and blood transfusion in multiple-level spine surgery from six RCT studies and five retrospective studies (Zhang et al., 2019). This study demonstrated that the administration of TXA can effectively decrease intraoperative blood loss and blood loss in spinal surgeries in contrast to the control group (*p*-value = 0.004) (Zhang et al., 2019). In addition, this study showed that the TXA group could maintain higher levels of postoperative hemoglobin when compared to the control group (*p*-value 0.009) (Zhang et al., 2019).

Goobie et al. studied the effect of higher TXA doses in 111 adolescent patients undergoing spinal surgery due to idiopathic scoliosis (Goobie et al., 2018). Patients were randomized into two groups: TXA (loading dose of 50 mg/kg after anesthesia induction followed by a maintenance dose of 10 mg/kg/h) or placebo (Goobie et al., 2018). The TXA group experienced a significantly lower amount of intraoperative bleeding (per spine level, rate per hour and total) when compared to placebo (*p* = 0.01, *p* < 0.001, and *p* = 0.02 respectively) (Goobie et al., 2018). Moreover, placebo was associated with a significant increase in postoperative bleeding when compared to the TXA group (645 ± 318 ml vs. 498 ± 228 respectively, *p* = 0.009) (Goobie et al., 2018). In addition, the relative risk of relevant blood loss (defined by authors as >20 ml/kg) was increased by 2.1 in patients receiving placebo with no thromboembolic events or seizures reported in any of the groups (Goobie et al., 2018). Nevertheless, in a series of 100 cases of patients undergoing complex spinal surgery and receiving a loading IV TXA dose of 50 mg/kg and a maintenance dose of 5 mg/kg/h, Lin et al. reported an incidence of 3% of thromboembolic events potentially related to TXA administration (Goobie et al., 2018). One patient (n = 1) experienced pulmonary embolism (PE) whereas deep vein thrombosis (DVT) was diagnosed in two patients. No myocardial infraction, seizures, stroke or renal failure was reported (Goobie et al., 2018).

This retrospective study demonstrated a higher portion of PE with TXA (2 subjects (4.3%) vs. 0%, *p* = 0.0487, respectively). When analyzing thrombotic complications together (PE and DVT) there was no difference between TXA and control. We believe this is likely due to the relatively small size of this study.

An essential limitation of this observational study was its retrospective design and single-center population. Therefore, the high variability of the data limited the number of eligible subjects for data analysis. Secondly, due to the retrospective nature of this study, the doses of loading and maintenance dose were not standardized and were instead at the surgeon’s discretion, leading to variability in dosing and possible influence over the results. Thirdly, as described by several studies with similar patient population, blood loss is also affected by other variables, such as the number and type of osteotomies performed (posterior column osteotomy, pedicle subtraction osteotomy or vertebral column resection) and the number and type of interbody fusions (posterior or transforaminal) performed (Johnson et al., 1989; Mirza et al., 2008; Naik et al., 2017). Fourthly, the number of subjects included in the data analysis for each group was not similar, resulting in 42 subjects in the control group and 34 in the TXA group. Lastly, this study was not powered to assess the hypothesis that the administration of IV TXA could decrease EBL and/or RBC units transfused.

## Conclusion

The results from the statistical analysis of this study suggest that the prophylactic use of intraoperative IV TXA during multilevel spinal fusion surgery could reduce intraoperative EBL and RBC unit transfusion requirements. Due to associated limitations of the retrospective design of this study, our findings should be corroborated in a prospective randomized dose-response study for further evaluation of the efficacy of standardized doses of TXA administration in this patient population.

## Data Availability Statement

The raw data supporting the conclusions of this article will be made available by the authors, without undue reservation.

## Ethics Statement

The studies involving human participants were reviewed and approved by The Office of Responsible Research Practices—The Ohio State University. Written informed consent for participation was not required for this study in accordance with the national legislation and the institutional requirements.

## Author Contributions

Study conception and design: AU, AT, ME-V, JF-D, BM, AG, SV, SB. Acquisition of data: AU, AT. Analysis and interpretation of data: AU, AT, MA-R. Drafting of manuscript: AU, AT, MA-R, ME-V, JF-D. Critical revision: AU, AT, ME-V, JF-D, BM, MA-R, AG, SV, SB.

## Conflict of Interest

The authors declare that the research was conducted in the absence of any commercial or financial relationships that could be construed as a potential conflict of interest.

## Abbreviations

**Abbreviations:** ABT, allogeneic blood transfusions; ASA, American Society of Anesthesiology; CONSORT, Consolidated Standards of Reporting Trials; CI, confidence interval; DVT, deep vein thrombosis; EBL, estimated blood loss; ICU, intensive care unit; IV, intravenous; RBCs, red blood cells; FFP, fresh frozen plasma; TXA, tranexamic acid; PACU, post-anesthesia care unit; PE, pulmonary embolism.
